# Profound sympathetic neuropathy in the bone marrow of patients with acute myeloid leukemia

**DOI:** 10.1038/s41375-023-02104-7

**Published:** 2023-12-08

**Authors:** Iryna Kovtun, Malte von Bonin, Liliia Ibneeva, Julia Frimmel, Jan Moritz Middeke, Desiree Kunadt, Lisa Heberling, Manja Wobus, Martin Bornhäuser, Tatyana Grinenko

**Affiliations:** 1 Institute for Clinical Chemistry and Laboratory Medicine, University Hospital Carl Gustav Carus and Faculty of Medicine, TU Dresden, Dresden, Germany; 2 Department of Internal Medicine I, University Hospital Carl Gustav Carus, TU Dresden, Dresden, Germany; 3 German Cancer Consortium (DKTK), Partner Site Dresden, and German Cancer Research Center (DKFZ), Heidelberg, Germany; 4 National Center for Tumor Diseases (NCT/UCC), Dresden, Germany; 5 Shanghai Institute of Hematology, State Key Laboratory of Medical Genomics, National Research Center for Translational Medicine at Shanghai, Ruijin Hospital Affiliated to Jiao Tong University School of Medicine, Shanghai, China

**Keywords:** Acute myeloid leukaemia, Cell biology, Acute myeloid leukaemia

## To the Editor:

Acute myeloid leukemia (AML) is a malignancy with a poor prognosis. The expansion of leukemic cells is associated with the impairment of normal hematopoiesis, which can lead to severe morbidity in affected patients. Recent studies suggested that leukemic cells can remodel the bone marrow (BM) microenvironment, the so-called BM niche, creating permissive conditions favoring leukemic stem cell expansion over normal hematopoietic stem and progenitor cells (HSPCs) [1]. BM niche is a complex three-dimensional (3D) structure that provides factors necessary for the maintenance, survival, and differentiation of healthy HSPCs [2]. Most of the BM niche studies were performed using murine models. Mouse BM niche is defined based on the anatomical localization of different cell types and includes mesenchymal stromal cells (MSCs), endothelial cells, macrophages, lymphocytes, and megakaryocytes [2]. In addition, sensory and sympathetic nerve fibers (SNF) penetrating the BM were described for the first time in animals more than 50 years ago [3]. Later, neuropeptide Y-expressing neurons and parasympathetic nerve fibers were detected in rat femurs [4]. The recent development of 3D imaging suggests that the sympathetic nervous system regulates balanced blood cell production [5], HSPC migration [6], and regeneration of hematopoiesis in response to genotoxic stresses [7] in murine models. Sensory signaling has been reported to modulate the functionality of MSCs and osteoclasts in murine BM [4]. In the case of AML, it was recently shown in a murine model generated by retroviral infection of HSPCs with MLL-AF9 that functional SNFs were disrupted around arterioles, leading to the expansion of MSCs and endothelial cells [8]. Therefore, it is reasonable to hypothesize that SNFs potentially can also regulate hematopoiesis in human BM.

The most studied human BM niche component is MSCs, as they play a central role in the control of HSPC fate by direct interaction and through the secretion of soluble factors [2]. It was shown that MSCs from AML patients have a decreased clonogenic capacity and proliferate less than MSCs from healthy donors in vitro. However, it is still unknown whether MSCs upon malignancy have tumor-promoting or tumor-suppressing effects. Several studies suggested that MSCs have the ability to form a cancer stem cell niche in xenograft mouse models and promote leukemia cell growth. At the same time, anti-inflammatory cytokines secreted by MSCs could cause cell cycle arrest of cancer cells, thus inhibiting tumor growth [1]. Furthermore, in mouse models of AML and myeloproliferative neoplasm, it has been shown that leukemia clones trigger damage of SNFs, which consequently affects the number of MSCs and endothelial cells in the BM, leading to suppression of normal hematopoiesis with a concomitant proliferation of mutant cells [8, 9].

Despite detailed characterization of murine BM microenvironment in steady state and tumor models, the 3D architecture of healthy and leukemia human BM niche remains underexplored due to the difficulty of obtaining BM biopsies, as many clinics have shifted from performing biopsies for diagnosis to primarily using BM aspirates. Thus, valuable information about the human BM niche, which cannot be obtained from a BM aspirate alone, is missing. This study aimed to analyze the human BM niche 3D architecture, including MSC distribution and sympathetic innervation in AML patients’ BM at diagnosis, during, and after cytotoxic therapy (CT).

**Fig. 1:**
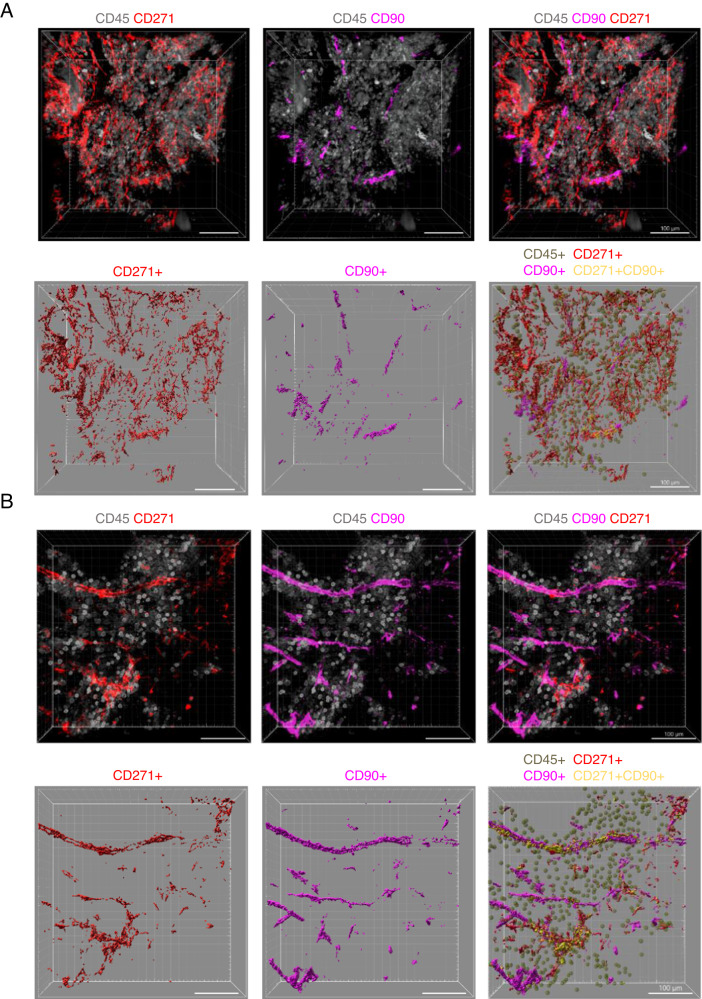
Distribution of MSCs in human BM. **A**, **B** Original representative z-stack confocal images from human BM biopsy samples obtained from patients without bone marrow involvement stained with CD271 (red), CD90 (pink), CD45 (gray) antibodies (top), and Imaris representation of the 3D BM architecture (bottom). CD45+ hematopoietic cells (dark green), CD45– CD271+ CD90– (red), CD45– CD271– CD90+ (pink), and CD45– CD271+ CD90+ MSCs (yellow). Images were acquired with 2 μm intervals to approximately 200- to 300 μm depths throughout the BM tissue, 5–7 images per sample.

To visualize the human BM niche architecture, we used 2-photon multicolor confocal microscopy, which allows us to penetrate the biopsy samples up to 300 μm deep with z-stack intervals of 2 μm. First, we investigated the distribution of MSCs. Until now, the studies of human MSCs remain very heterogeneous because of the lack of unique and definitive cellular markers for their identification. While some researchers consider CD271 (low-affinity nerve growth factor receptor) as a pan-MSC marker [10], others use CD90 (Thy-1) for clonogenic MSCs [11] and suggest that most of them do not express CD271 [12]. The discrepancy in the MSC phenotype probably comes from the different procedures for MSC isolation or different culture conditions, as most investigations were done in vitro. In addition, the precise localization of MSCs expressing CD271 or CD90 in human BM remains unknown. Due to the difficulty of obtaining BM biopsy samples from healthy individuals, we used samples from three patients with lymphoma and one with tubular adenoma without BM infiltration before any treatment as a control BM (*n* = 4, age: median = 56 years (range: 38–61), sex: male = 3, female = 1) (Suppl. Table 1). We stained trephine biopsy samples with anti-CD90 and CD271 antibodies to investigate MSC distribution. We found that CD45- CD271+ cells, which localize along the long cylindrical structures typical for BM vessels, express CD90, while most CD45– CD271+ CD90– cells are distributed irregularly in cellular parenchyma (Fig. 1A, B; Fig. S1 and Suppl. Video 1). Therefore, we suggest that CD271 can be used as a pan-MSC marker, while CD90 most probably defines perivascular cells.

**Fig. 2:**
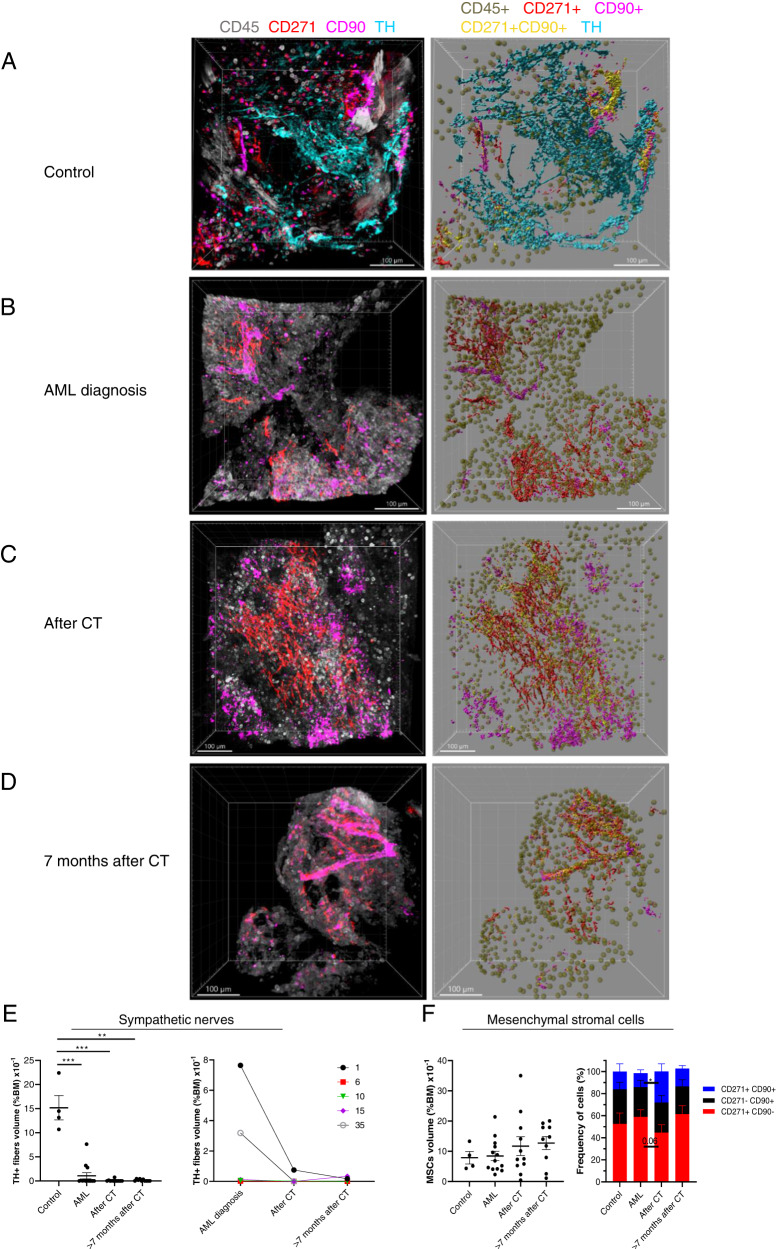
AML induces sympathetic nerve fibers injury. **A**–**D** Original representative z-stack confocal images of BM biopsy samples from **A** control patients’ BM, patients **B** at primary AML diagnosis, **C** 15–21 days after CT, and **D** > 7 months after CT (left). The right panels display Imaris extracted images: CD45+ hematopoietic cells (dark green), TH+ SNFs (cyan), CD45– CD271+ CD90– (red), CD45– CD271– CD90+ (pink), and CD45– CD271+ CD90+ MSCs (yellow). Samples were stained with anti-TH (cyan), CD271 (red), CD90 (pink), and CD45 (gray). Images were acquired with 2 μm intervals to approximately 200- to 300 μm depths throughout the BM tissue, 5–7 images per sample. **E** Quantification of SNFs in BM from control patients (*n* = 4), from patients at AML diagnosis (*n* = 13), after CT (*n* = 11), and > 7 months after CT (*n* = 10) (left). The right panel represents SNF percentage in BM (ratio between the SNF volume and total volume from all pictures for each patient x 100 %) over time for the same patient (*n* = 5). **F** Quantification of MSC densities (the ratio between the MSC volume and total volume from all pictures for each patient x 100 %) in control BM (*n* = 4), from patients at AML diagnosis (*n* = 13), after CT (*n* = 11), and > 7 months after CT (*n* = 10) (left). Composition of CD45– CD271+ CD90+, CD45– CD271– CD90+, and CD45– CD271+ CD90– MSCs in human BM (right). Each dot represents a single patient sample. Data represented as mean +/– S.E.M. *p < 0.05, *p < 0.01, ***p < 0.001 determined by unpaired Mann–Whitney test.

To investigate the distribution of SNFs in human BM, we used tyrosine hydroxylase (TH) staining as a marker for functional SNFs [8, 9] (Fig. 2A; Fig. S2 and Suppl. Video 2). We found that TH+ SNFs in human BM do not show a corkscrew-shaped appearance due to wrapping around blood vessels like it was shown in murine BM [5, 7, 8, 13] but are distributed irregularly in the parenchyma (Fig. 2A and Fig. S2), indicating that findings and conclusions drawn from research conducted on mice may not be uniformly translated to human BM niche due to inherent biological differences between the two species.

Next, we analyzed whether AML development and CT change the density of MSCs and TH+ SNFs in patients’ BM. We collected trephine biopsy samples during routine diagnostic procedures from 25 AML patients (age: median = 64 years (range: 22–81), sex: male = 14, female = 11, ELNRisk 2017 categories: adverse = 16, intermediate = 2, favorable = 7) (Suppl. Table 1). The density of TH+ SNFs was significantly reduced in the BM of AML patients already at the primary diagnosis compared to controls (Fig. 2A–E and Fig. S3). The density of residual TH+ SNFs did not correlate with neither age nor blast frequency at the primary diagnosis and was dramatically reduced even in the BM of patients with low blast frequency (10–20 %) (Fig. S4A, B). However, we noticed that 3 out of 6 patients from favorable or intermediate risk categories had higher TH+ SNF density (2.7–7.6 × 10^−1^ % BM volume) compared with those from the adverse category (in all samples below 0.01 × 10^−1^ % BM volume) (Fig. S4C and Suppl. Table 1). Importantly, we found that the drugs used for induction CT (Suppl. Table 1) reduce the density of TH+ SNFs in BM even further compared to primary diagnosis samples (Fig. 2B–E; Fig. S3 and Suppl. Table 1), which is in agreement with murine models of cytotoxic treatment with vincristine and cisplatin [7]. Moreover, TH+ sympathetic fibers did not restore even seven months after CT (Fig. 2D, E and Fig. S3).

It has been shown that sympathetic neuropathy in BM induces changes in MSC number in murine leukemia models [8, 9]. However, we found that MSC population density and composition were not changed in the BM of AML patients despite the severe reduction in sympathetic innervation compared to controls at any analyzed time points (Fig. 2A–F and Fig. S5).

Therefore, for the first time in human biopsy material, we visualized MSCs and TH+ SNFs in control and AML BM. Our study reveals the detrimental and persistent effect of invasive leukemic growth on the sympathetic neural component of the human BM niche, which can lead to long-lasting hematological dysfunction. Since sympathetic reinnervation after solid organ transplantation has been associated with improved organ function [14], further investigation should be performed to validate whether the restoration of functional TH+ SNFs can be a therapeutic goal in patients with AML.

### Supplementary information

Supplemental material

Suppl. Table 1

Suppl. Video 1

Suppl. Video 2

## Data Availability

For original data please contact Tatyana.grinenko@uniklinikum-dresden.de.
