# Supplementary material for: Profound sympathetic neuropathy in the bone marrow of patients with acute myeloid leukemia
Source: Leukemia. 2023 Dec 8;38(2):393–7. doi: 10.1038/s41375-023-02104-7 (PMC10844069; doi:10.1038/s41375-023-02104-7)
Supplement: Supplementary file 1 — Supplemental material [file 41375_2023_2104_MOESM1_ESM.docx]

**Supplementary materials**

**Patients**

Trephine biopsy samples were collected from routine diagnostic procedures from 29 patients with hematologic malignancy after informed consent. Samples from three patients with lymphoma and one with tubular adenoma without BM infiltration before any treatment were used as a control BM n=4 (age: median=56 years (range: 38-61), sex: male=3, female=1). Samples from AML patients n=25 (age: median=64 years (range: 22-81), sex: male=14, female=11, ELNRisk 2017 categories: adverse=16, intermediate=2, favorable=7); primary diagnosis only (n=8), after induction CT only (n=12), diagnosis and follow up after CT (n=5). Detailed information about patients can be found in Supplemental Table 1. The study was approved by the ethical board of the TU Dresden (BO-EK-179032021).

**Immunostaining and imaging data acquisition**

For immunofluorescence staining, samples were fixed in 4% PFA for 1-2 h RT followed by blocking with 20% NGS (MP Biomedicals) in 0.5% Triton X-100 / PBS for 3 h and incubation with rabbit anti-tyrosine hydroxylase (P21962, Invitrogen), CD90 APC (A15726, Invitrogen), and CD271 PE (12-9400-42, eBioscience) antibodies in 2% NGS, 0.1% Triton X-100 / PBS for 72 h. After washing with PBS, samples were incubated with anti-rabbit BV421 (406410, Biolegend) and CD45 BV421 or FITC (368522 or 304005, Biolegend) antibodies for 72 h. Three-dimensional (3D) imaging data were acquired using an upright Leica SP8 microscope equipped with a Coherent Vision II multiphoton laser. Z-Stacks with 2μm intervals to 200μm-300μm depths throughout the BM tissue were acquired at 1024 × 1024 sampling with a voxel size of 0.433 x 0.433 x 2.0μm using a 25x/0.95NA water immersion objective (Leica). BV421 and APC were excited at 860nm and detected from 415 to 485nm and 665-705nm, respectively, on non-descanned PMTs. PE was excited with 1060nm and detected from 578 to 645nm on a non-descanned HyD. 5-7 images per sample were acquired.

Analysis was performed using Imaris 9.9.1 software (Bitplane/Oxford Instruments). The “surface” function was used to quantify cellular volumes. Signals from all channels were combined to create a total cellular volume; “Channel Arithmetics” function was used to separate different populations of MSCs based on CD90 and CD271 expression and the absence of CD45 expression. Density was calculated as ratio between the specific cellular volume from all datasets and total volume from all datasets for each patient.

Data are presented as mean ± SEM. Statistical analyses based on the unpaired Mann-Whitney test were performed using the Prism 8.2.1 software (GraphPad). P-value < 0.05 was considered statistically significant.


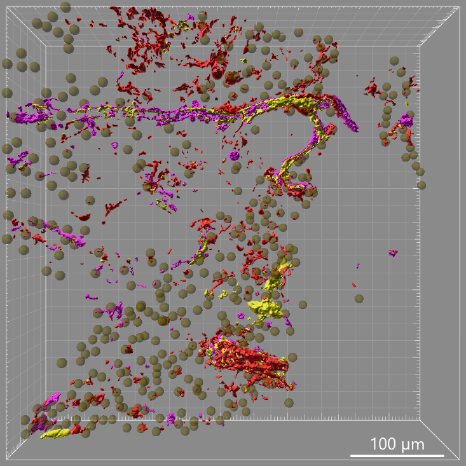

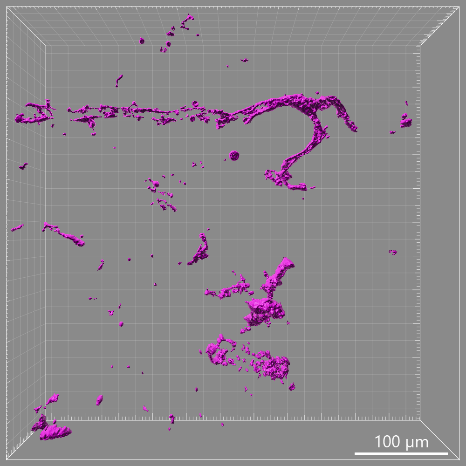

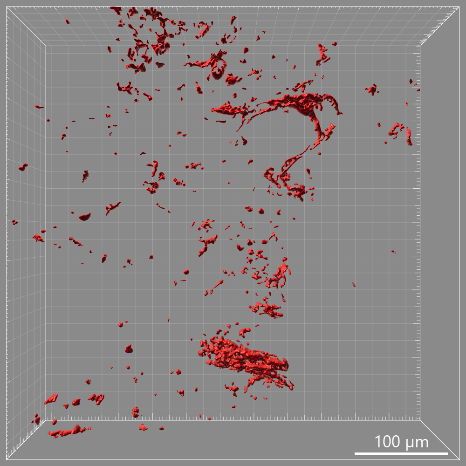

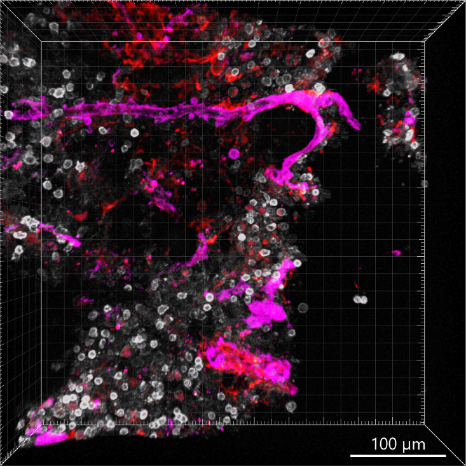

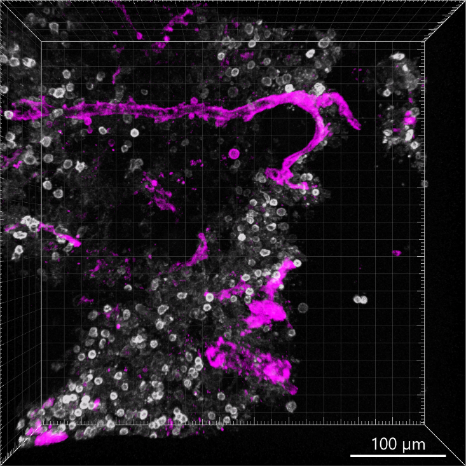

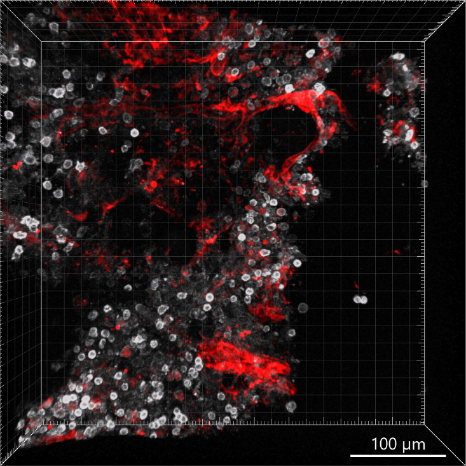


Fig. S1

CD45 CD271 CD90

CD271+

CD90+

CD45 CD271

CD45 CD90

CD45 CD271 CD90

CD45+ CD271+

CD90+ CD271+CD90+

**Fig. S1. Distribution of MSCs in human BM.** Trephine biopsy specimens of control BM were stained with CD271 (red), CD90 (pink), and CD45 (grey) antibodies (top). The bottom panels display Imaris representation of the spatial distribution of MSCs (CD45- CD271+ CD90- (red), CD45- CD271- CD90+ (pink), CD45- CD271+ CD90+ (yellow)) and CD45+ hematopoietic cells (dark green).

Fig. S2

A


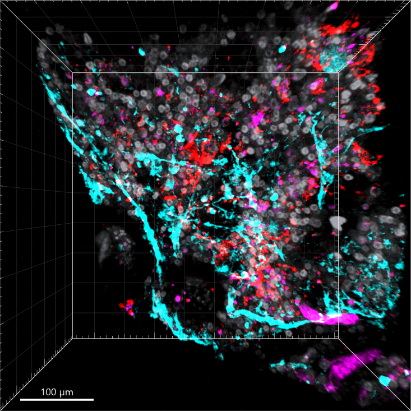

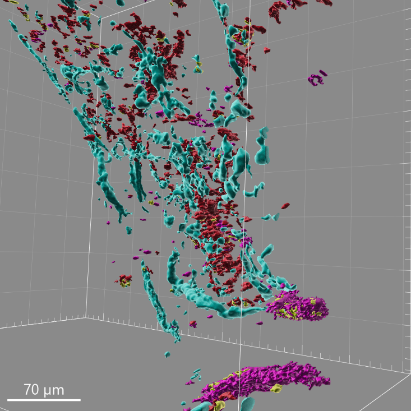

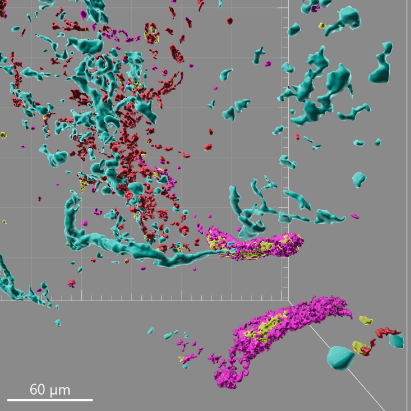

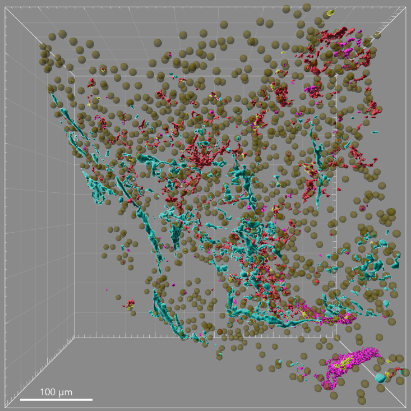


CD45+ CD271+ CD90+ CD271+CD90+ TH+

CD45 CD271 CD90 TH

CD45+ CD271+ CD90+ CD271+CD90+ TH+

CD45 CD271 CD90 TH


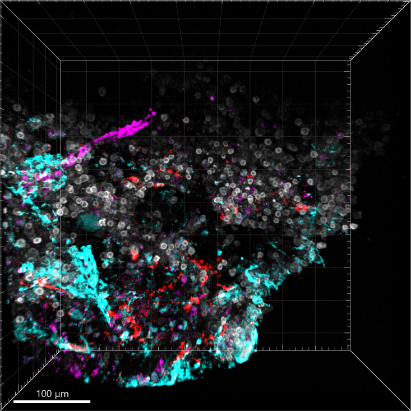

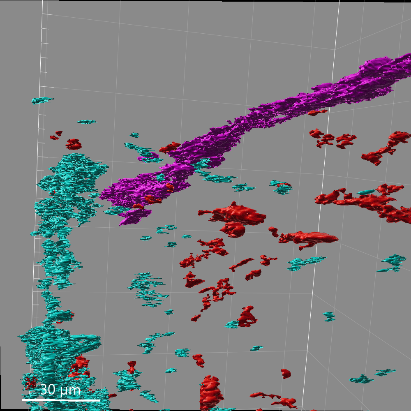

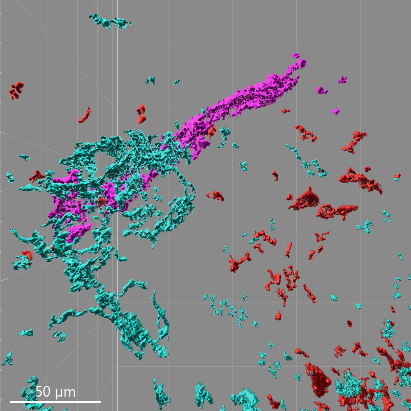

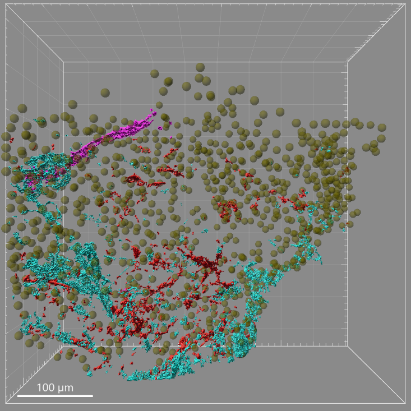


B

**Fig. S2. Distribution of sympathetic nerve fibers in human BM.**

**A-B** Top left panels represent original images of BM from two control patients’ BM stained with anti-TH (cyan), CD271 (red), CD90 (pink), CD45 (grey) antibodies. Top rights panels display Imaris representation of images. The bottom panels represent magnified zoom-in areas within the regions defined by the rectangles showing that TH+ SNFs are not wrapped around vessels but localize irregularly.

Fig. S3


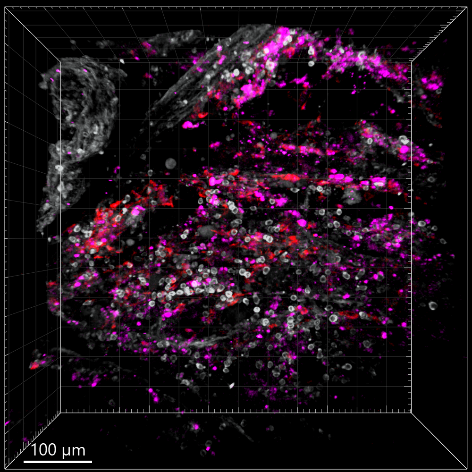

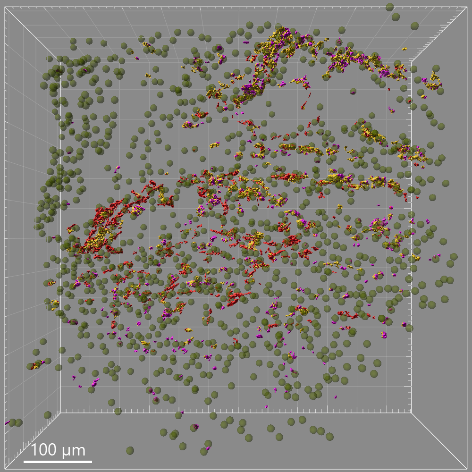

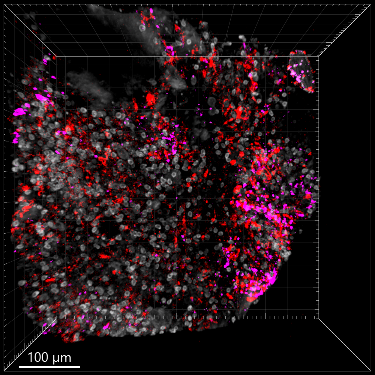

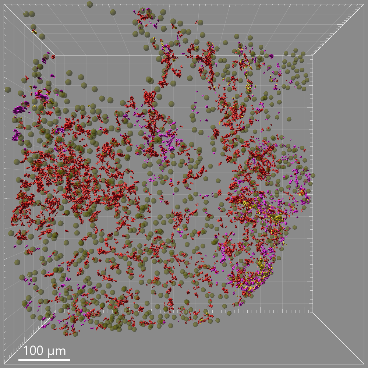

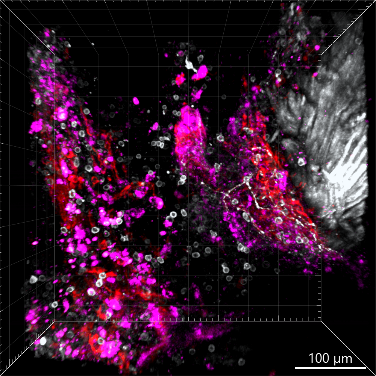

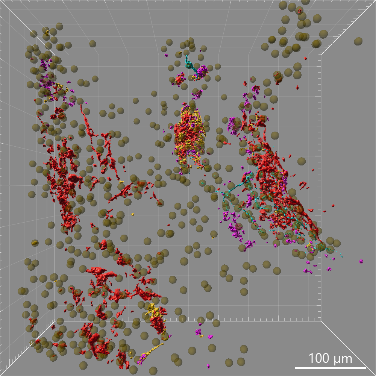


C

B

A

CD45+ CD271+ CD90+ CD271+CD90+ TH+

CD45 CD271 CD90 TH

7 months after CT

After CT

AML diagnosis

**Fig. S3. Sympathetic nerve fibers are not restored in BM of AML patients after cytotoxic therapy.**

**A-C** Samples from one individual patient were collected at three time points: **(A)** at AML diagnosis, **(B)** 15 days after CT, and **(C)** at a seven-month follow-up interval. Left: original images stained with anti-TH (gray), CD271 (red), CD90 (pink), CD45 (grey) antibodies; right: Imaris representation of TH+ SNFs (cyan), MSCs (CD45- CD271+ CD90- (red), CD45- CD271- CD90+ (pink), CD45- CD271+ CD90+ (yellow)) and CD45+ hematopoietic cells (dark green).

C

B

A

Fig. S4

**Fig. S4. At the diagnosis stage, the density of TH+ sympathetic nerve fibers in the BM of AML patients did not correlate with blast frequency and age.**

**A-C** Scatter plots demonstrating the correlation between TH+ fibers densities and **(A)** the percentage of blasts, **(B)** age and **(C)** ELNRisk 2017 categories within BM samples at AML diagnosis stage. Each data point corresponds to an individual patient sample. Favorable (fav), intermediate (int), and adverse (adv).

C

B

A

Fig. S5

**Fig. S5. The density of different MSC populations in BM of AML patients.**

**A-C** Quantification of three distinct CD45- MSC populations’ densities in control BM, samples from primary AML diagnosis, after induction CT, and >7 months after CT.

**Supplemental video 1**

The 3D animation of human BM. Spatial visualization of CD45- CD271+ CD90- (red), CD45- CD271- CD90+ (pink), CD45- CD271+ CD90+ MSCs (yellow), and CD45+ hematopoietic cells (dark green).

**Supplemental video 2**

The 3D animation of human BM innervation. Spatial visualization of TH+ nerve fibers (cyan), CD45- CD271+ CD90- MSCs (red), CD45- CD271- CD90+ (pink), CD45- CD271+ CD90+ MSCs (yellow), and CD45+ hematopoietic cells (dark green).
